# Brain activity during traditional textbook and audiovisual‐3D learning

**DOI:** 10.1002/brb3.1427

**Published:** 2019-09-30

**Authors:** Jesus Pujol, Laura Blanco‐Hinojo, Gerard Martínez‐Vilavella, Lucila Canu‐Martín, Anna Pujol, Víctor Pérez‐Sola, Joan Deus

**Affiliations:** ^1^ MRI Research Unit Department of Radiology Hospital del Mar Barcelona Spain; ^2^ Centro Investigación Biomédica en Red de Salud Mental, CIBERSAM G21 Barcelona Spain; ^3^ Institute of Neuropsychiatry and Addictions Hospital del Mar, IMIM Barcelona Spain; ^4^ Department of Psychobiology and Methodology in Health Sciences Autonomous University of Barcelona Barcelona Spain

**Keywords:** education, functional MRI, learning, memory, prefrontal cortex

## Abstract

**Introduction:**

Audiovisual educational tools have increasingly been used during the past years to complement and compete with traditional textbooks. However, little is known as to how the brain processes didactic information presented in different formats. We directly assessed brain activity during learning using both traditional textbook and audiovisual‐3D material.

**Methods:**

A homogeneous sample of 30 young adults with active study habits was assessed. Educational material on the subject of Cardiology was adapted to be presented during the acquisition of functional MRI.

**Results:**

When tested after image acquisition, participants obtained similar examination scores for both formats. Evoked brain activity was robust during both traditional textbook and audiovisual‐3D lessons, but a greater number of brain systems were implicated in the processing of audiovisual‐3D information, consistent with its multisource sensory nature. However, learning was not associated with group mean brain activations, but was instead predicted by distinct functional MRI signal changes in the frontal lobes and showed distinct cognitive correlates. In the audiovisual‐3D version, examination scores were positively correlated with late‐evoked prefrontal cortex activity and working memory, and negatively correlated with language‐related frontal areas and verbal memory. As for the traditional textbook version, the fewer results obtained suggested the opposite pattern, with examination scores negatively correlating with prefrontal cortex activity evoked during the lesson.

**Conclusions:**

Overall, the results indicate that a similar level of knowledge may be achieved via different cognitive strategies. In our experiment, audiovisual learning appeared to benefit from prefrontal executive resources (as opposed to memorizing verbal information) more than traditional textbook learning.

## Graphical abstract

We report the neural correlates of traditional textbook and audiovisual learning. A similar level of knowledge may be achieved with both educational formats. Audiovisual learning benefits from executive resources more than from verbal memory.

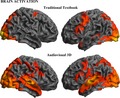

## 1 INTRODUCTION

Audiovisual educational tools have increasingly been used during the past years to complement and compete with traditional textbooks (Jarvin, 2015; Lewis, 2003; Prakash, Muthuraman, & Anand, 2017; Trelease, 2016). However, little is known as to how the brain processes didactic information presented in different formats. Some work has been done to characterize the differences in the evoked brain response to verbal information presented visually or aurally (e.g., Buchweitz, Mason, Tomitch, & Just, 2009; Venezia et al., 2017), and a recent study has explicitly compared brain activity during the viewing of complex scenes (movies) and reading its screenplay text (scripts) (Tikka, Kauttonen, & Hlushchuk, 2018). Nevertheless, there are no studies directly investigating brain activity predicting the learning achieved during both audiovisual and text lessons.

We used functional MRI (fMRI) to assess brain activity during lesson learning in a homogeneous sample of young adults with active study habits. Educational material on the subject of Cardiology was adapted for presentation within the constrictions of the MRI experimental environment. The traditional textbook task involved the visual presentation of text and high‐quality illustrations. In the audiovisual version, identical verbal information was presented via audio, and visual stimuli involved a dynamic 3D illustration of the presented educational concepts.

We firstly aimed to identify differences in evoked brain activity between both traditional textbook and audiovisual‐3D versions. We presumed that, as a group effect, the audiovisual‐3D format would be able to capture a greater variety of brain elements due to its multisource sensory nature. Nevertheless, brain group activations may not accurately reflect the neural correlates of learning (Foulkes & Blakemore, 2018; Friedman & Miyake, 2017; Seghier & Price, 2018), to the extent that large individual differences typically exist in learning complex information (Jonassen & Grabowski, 1993). Therefore, a correlation analysis was also conducted with the aim of specifically identifying brain activity predicting acquired knowledge measured by a multiple‐choice examination.

According to outstanding conceptions on the neural bases of learning (Fuster, 2001; Miller & Cohen, 2001; Shallice & Cipolotti, 2018; Wager & Smith, 2003; Wood & Grafman, 2003), we hypothesized that knowledge acquisition would significantly depend on the participation of prefrontal lobe structures. Thus, individual differences in examination scores would correspond to a different involvement of the prefrontal cortex controlling executive resources during learning. A selection of cognitive tests was also used to characterize adopted cognitive learning strategies. The assessed cognitive domains included working memory, verbal memory, visual memory, incidental visual memory, vocabulary, and cognitive processing speed.

## 2 METHODS

### 2.1 Study participants

The final study sample involved a total of 30 university students, all of whom were studying their final year of Psychology or a postgraduate Psychology master. The selected group was therefore maximally homogeneous in terms of learning capabilities. The group included 15 men and 15 women with a mean (±*SD*) age of 24.2 (±3.9) years, range 21–39 years. Participants were not paid for their contribution.

The study was conducted in accordance with the principles expressed in the Declaration of Helsinki. The protocol was approved by the Autonomous University of Barcelona's Ethics Commission on Animal and Human Experimentation (CEEAH: 4217). Written informed consent was obtained from all participants.

### 2.2 The learning stimuli

**Figure 1:**
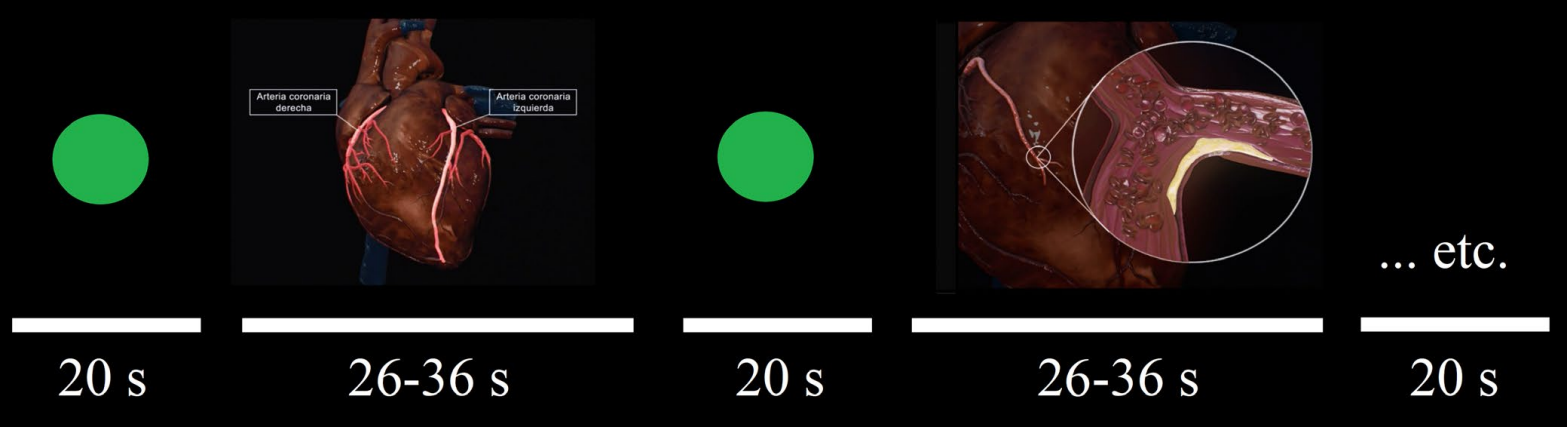
Illustration of the stimuli presented during the fMRI experiment for the audiovisual‐3D format, coronary artery content. The paradigm involved a baseline condition with six point‐fixation blocks and a test condition with six blocks each presenting a lesson fragment. An identical presentation sequence was used for the traditional textbook format

The lessons to be learned were based on the subject of Cardiology, with which the Psychology students were unfamiliar. Two similarly structured lessons were devised, based on coronary arteries and heart valves. Both lessons were adapted to traditional textbook and audiovisual‐3D formats. The duration of the lessons was 3 m and 14 s, and each (coronary arteries and heart valves in both traditional textbook and audiovisual‐3D formats) was segmented into six fragments of 26–36 s. Lessons in traditional textbook format were presented using PowerPoint (PPT) slides, whilst the audiovisual‐3D version was displayed in a movie format. In each case, the six fragments were presented interleaved with a baseline condition, in which a green fixation point was presented for 20 s. Therefore, the experiment involved two fMRI runs of 5 m and 14 s (traditional textbook and audiovisual‐3D presented in a counterbalanced order), each including a baseline condition with six point‐fixation blocks alternated with six test‐condition blocks presenting a lesson fragment (Figure 1). The Videos S1 and S2 (mp4 files), show a fragment of the coronary artery lesson in both textbook and audiovisual formats. All the didactic material was developed in 3D Tech Omega Zeta SL (Barcelona, Spain) by personnel specialized in developing 3D educational platforms.

### 2.3 Procedure

Participants were previously given instructions concerning fMRI testing procedures and the need to remain still during the acquisition. They were specifically requested to try and understand the didactic material and not simply memorize the presented items. Each participant received one lesson (coronary arteries or heart valves) in the traditional textbook format and another (coronary arteries or heart valves) in the audiovisual‐3D format. Therefore, each participant was exposed to both formats, but the lesson presented was always different. The order of both format (traditional textbook or audiovisual‐3D) and topic (coronary arteries or heart valves) was fully counterbalanced across participants with a pseudorandom allocation.

Following the fMRI scans, knowledge acquisition was assessed in each participant by means of a multiple‐choice test including 10 questions on each topic. As an interference task between the fMRI and the examination, the participants were required to read a text (two stories from the Wechsler Memory Scale, 1987). The time elapsed between the presentation of the Cardiology lessons and the formal examination ranged from 25 to 35 m.

In addition, a 101‐point numerical rating scale (NRS) was used for the participants to rate the extent to which the presented didactic material was enjoyable, for both formats. The participants were also required to express their preference for either the traditional format or the audiovisual format. Finally, the selected cognitive tests were administered in a single session the same day of the MRI experiment.

### 2.4 Selected cognitive assessment

#### 2.4.1 Working memory

The Digit Span subtest of the Wechsler Adult Intelligence Scale (WAIS) was used as a conventional measure of working memory (Wechsler, 2008). In this test, the participant is required to repeat digits forward and backward in order, immediately after their oral presentation by the examiner. The overall age‐appropriate normed (i.e., standardized to have a mean of 10 and a standard deviation of 3) score, combining forward and backward performance, was used in our analyses.

#### 2.4.2 Verbal memory

The classic Verbal Paired Associates test from the Wechsler Memory Scale (Wechsler, 1987) was used. The test evaluates verbal memory for associated word pairs. After 10 word pairs are read to the participant, the first word of each pair is then read and the subject is requested to provide the corresponding associated word. The test involves three trials based on the same list presented in different order. Age‐appropriate normed scores were used.

#### 2.4.3 Visual memory

The Visual Reproduction subtest from the Wechsler Memory Scale (Wechsler, 1987) was used. In this test, a series of three geometric pictures of increasing complexity is shown, one at a time, for 10 s each. Immediately after the presentation of each picture, the participant is asked to draw it from memory. Each picture is scored by adding up the number of correctly reproduced image components. Age‐appropriate normed scores were used.

#### 2.4.4 Processing speed and incidental visual memory

The Digit Symbol subtest of the Wechsler Adult Intelligence Scale (WAIS) was used as a conventional measure of speed in visuoperceptive processing (Wechsler, 2008). The participant is required to copy (in the spaces provided below the rows of numbers) the symbols matching each number on the basis of a key located at the top of the page. The participant is given a total of 120 s to copy the symbols as quickly as possible. The number of correct symbols, depicted within the allotted time, was measured and subsequently converted into normed scores.

The Digit Symbol test also served to measure Incidental Visual Memory, using the form Digit Symbol—Incidental Learning, pairing procedure. Immediately after the Digit Symbol test, the participant is required to complete two series of Digit Symbol items with not access to the code key. The participants are never informed that their memory would be tested. Direct scores (i.e., the number of completed items) were converted into percentile scores.

#### 2.4.5 Vocabulary

The Vocabulary subtest of the Wechsler Adult Intelligence Scale (WAIS) was used (Wechsler, 2008). The participant is required to define up to 30 words of increasing difficulty presented orally by the examiner. This test assesses both language and retrieval of information from long‐term memory. Direct scores (from 0 to 2 points for each word) were converted into normed scores.

### 2.5 Functional MRI

#### 2.5.1 Functional MRI acquisition

A Philips Achieva 3.0 Tesla magnet (Philips Healthcare), equipped with an eight‐channel phased‐array head coil and single‐shot echoplanar imaging (EPI) software, was used for the MRI assessment. Stimuli were presented using MRI‐compatible high‐resolution goggles and audio system (VisuaStim Digital System, Resonance Technology Inc.). Our stimuli presentation system did not include the “MR eye tracking” option.

Functional sequences consisted of gradient recalled acquisition in the steady state (time of repetition [TR], 2,000 ms; time of echo [TE], 35 ms; pulse angle, 70°) within a field of view of 240 × 240 × 128 mm, with a 64 × 64‐pixel matrix, and a slice thickness of 4 mm (interslice gap, 0 mm) and acquisition voxel size of 3.75 × 3.75 × 4 mm. A total of 32 interleaved slices were acquired to cover the whole brain. Each functional time series consisted of 157 consecutive image sets or volumes obtained over 5 m and 14 s. The first four (additional) image sets in each run were discarded to allow the magnetization to reach equilibrium. High‐resolution anatomical images were additionally obtained using a sagittal T1‐weighted three‐dimensional fast spoiled gradient (SPGR) sequence. A total of 160 slices were acquired with repetition time = 8.1 ms; echo time = 3.7 ms; flip angle = 8°, field of view = 240 × 240 × 160 mm; matrix size 256 × 256 pixels, in‐plane resolution = 0.94 × 0.94 mm^2^; and slice thickness = 1 mm.

##### Image preprocessing

Imaging data were processed using MATLAB version 2016a (The MathWorks Inc) and Statistical Parametric Mapping software (SPM12; The Wellcome Department of Imaging Neuroscience). Preprocessing involved motion correction, spatial normalization, and smoothing by means of a Gaussian filter (full‐width half‐maximum, 8 mm). Data were normalized to the standard SPM‐EPI template and resliced to 2 mm isotropic resolution in Montreal Neurological Institute (MNI) space. All image sequences were inspected for potential acquisition and normalization artifacts. At this stage, two participants were removed from an initial broader sample of 34 subjects as a result of air/bone‐related magnetic susceptibility artifacts, a third participant due to excessive motion (mean interscan motion of 1.9 mm), and a fourth subject showing incidental MRI findings (white matter hypersignal intensities).

##### Control of potential head motion effects

To control for the effects of head motion, we adopted the following approach: (a) time series were aligned to the first image volume in each participant using a least squares minimization and a 6‐parameter (rigid body) spatial transformation. (b) Six motion‐related regressors and their six derivatives were included in the first‐level (single‐subject) analysis. (c) Within‐subject, censoring‐based MRI signal artifact removal (scrubbing) (Power et al., 2014) was used to discard motion‐affected volumes. For each participant, interframe motion measurements (Pujol et al., 2014) served as an index of data quality to flag volumes of suspect quality across the run. At points with interframe motion >0.2 mm, we discarded the corresponding volume, together with the preceding volume, and the two succeeding volumes. Using this procedure, a mean of 2.7 (1%) volumes (range 0–20) was removed from the total of 314 analyzed combining the two functional MRI sequences obtained in each participant.

### 2.6 Statistical analysis

#### 2.6.1 Behavioral data

Paired Student's *t* test was used to compare mean differences within the study group in terms of behavioral ratings. A chi‐squared test was used to assess the relationships between categorical variables. Pearson's product–moment correlations were used to test the association between the participants' examination scores and performance in cognitive testing. Multiple regression analysis was used to predict examination scores with a combination of variables including cognitive testing and extracted measures of brain activity in regions of interest (i.e., frontal lobe regions showing significant correlation with examination scores; see below).

#### 2.6.2 Functional MRI data

To obtain individual maps of brain activity evoked during lesson presentation, a boxcar regressor was generated considering the six blocks of the baseline condition and the six blocks of the test condition and applying a hemodynamic delay of 4 s. The contrasts “baseline < test condition” (activation) and “baseline > test condition” (deactivation) were estimated for each participant. The resulting first‐level SPM contrast images were carried forward to group‐level random‐effects analyses. One‐sample *t* test designs were used to generate group activation maps. Paired *t* tests were used to compare brain activity evoked during learning using both traditional textbook and audiovisual‐3D formats.

In addition, voxel‐wise linear regression was used to test the association between examinations scores and evoked brain activity in the corresponding experiment (i.e., separately for the traditional textbook format and the audiovisual‐3D format). The results from this correlation analysis were reported in terms of both *t*‐values (SPM default) and Pearson's *r*‐values ($t=r/\sqrt{1-r^{2}/n-2}$). A primary analysis tested the correlation with brain activity evoked during the entire lesson. Further analyses tested the correlation at four different periods of 6 s (three frames), using data from the beginning, middle, and end of each lesson fragment and immediately (no time lap) after the lesson fragment. Data from regions showing significant correlation with examination scores were extracted for plotting purposes and to carry out seed analyses to identify co‐activated brain networks.

##### Seed analyses

As in our previous studies (Pujol et al., 2019, 2016), the seed region was defined as a 3.5‐mm radial sphere (sampling ~ 25 voxels in 2 mm isotropic space) centered in each identified brain region. This was performed using MarsBaR region of interest (ROI) toolbox in MNI stereotaxic space. Signals of interest were extracted by calculating the mean ROI value at each time point across the time series. To generate the seed maps, the signal time course of a selected seed region was used as a regressor to be correlated with the signal time course of each brain voxel to generate individual voxel‐wise statistical parametric maps of co‐activated regions. A high‐pass filter set at 128 s was used to remove low‐frequency drifts below ~0.008 Hz. In addition, we derived estimates of white matter, CSF, global brain signal fluctuations, and 12 motion‐related regressors to be included in the analyses as nuisance variables. The first level of our statistical analysis, therefore, involved the generation of single‐subject brain maps expressed as beta regression estimates. Single‐subject voxel‐wise functional connectivity maps were then included in second‐level (group) random‐effects analyses to test for group effects (one‐sample *t* tests).

##### Thresholding criteria

In whole‐brain analyses, clusters >1.26 ml (158 voxels) at a height threshold of *p* < .005 were considered, which satisfied the family‐wise error (FWE) rate correction of *p*_FWE_ < .05, according to Monte Carlo simulations. In analyses within the frontal lobe, the corresponding cluster size considered was 0.94 ml (118 voxels).

## 3 RESULTS

### 3.1 Behavioral results

#### 3.1.1 Subjective evaluation

The audiovisual‐3D format was considered by the participants to be significantly more enjoyable than the traditional textbook format (group mean score ± *SD*; 88.3 ± 10.9 vs. 69.3 ± 19.3; *t*_29_ = 5.0, and *p* = .00003). All 30 participants (100%) answered “yes” to the question “Would you use the 3D video format to complement the traditional method of study?” In the event of compulsory expression of preference, 21 of the 30 participants would choose the video format and nine the traditional textbook format for the future (χ^2^_1,_ *_N _*_= 30_ = 4.8; *p* = .028).

#### 3.1.2 Objective evaluation

Despite format preferences, the achieved learning was similar following both lessons. In the 10‐question examinations, the participants’ correct answer mean was 6.1 (*SD*, 2.3 and range, 1–10) when the didactic information was presented using the audiovisual‐3D format and 5.9 (*SD*, 1.8 and range 3–9) when using the traditional textbook format. The difference was not statistically significant *t*_29_ = 0.8, and *p* = .752). Noteworthy are the participants' wide examination score ranges denoting large individual differences in lesson learning.

Regarding the participants' poor success rate, three questions were identified as highly difficult (i.e., correct answers < 15% when presented using the traditional textbook format). Interestingly, when considering only such highly difficult questions (questions 1, 2, and 9 of the heart valve examination), better examination outcomes were obtained with the audiovisual‐3D format (33% of correct answers) than with the traditional textbook format (12% correct answers), with *t*_29_ = 2.4 and *p* = .024.

#### 3.1.3 Cognitive testing

**Figure 2:**
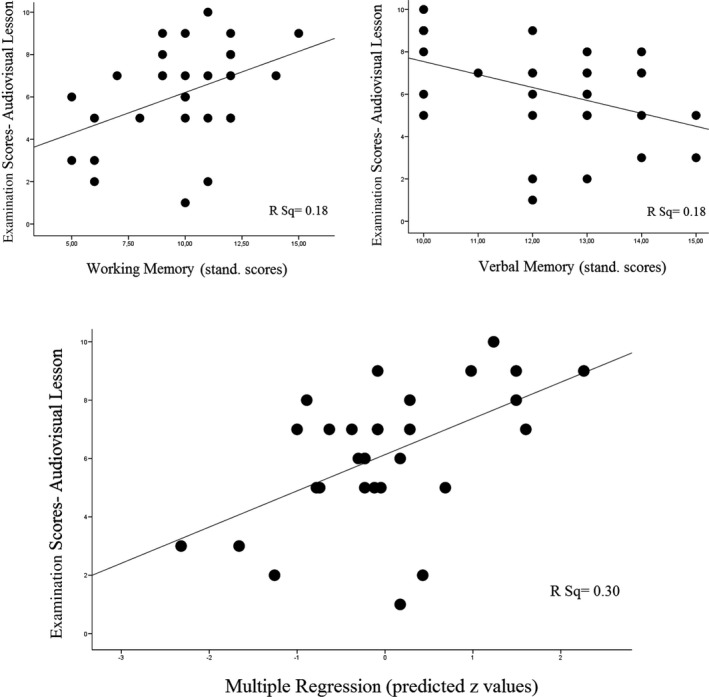
Plots showing the associations between examination scores from lessons presented with the audiovisual‐3D format and memory performance

Table 1 reports the participants' performance in selected memory and cognitive tests and the pattern of correlations with their scores on learning Cardiology lessons. Examination scores for lessons presented with the audiovisual‐3D format positively correlated with working memory and negatively correlated with verbal memory. Significantly, working memory and verbal memory performance jointly explained 24% of the audiovisual‐3D examination score variance when included in a multiple regression analysis (Multiple *R* = 0.54, *r* ^2^ = 0.30, adjusted *r* ^2^ = 0.24, *F*_2,27_ = 5.6, *p* = .009). Figure 2 illustrates such an association between memory domains and audiovisual examination scores.

**Table 1:** Cognitive testing (*N* = 30)

	Performance	Correlation with examination scores	
		Audiovisual‐3D	Traditional Textbook
	Mean ± *SD*	*r* (*p*)	*r* (*p*)
Working memory (normed scores)	9.8 ± 2.5	**.43 (.019)**	−.14 (.468)
Verbal memory (normed scores)	12.3 ± 1.6	**−.42 (.020)**	.34 (.066)
Visual memory (normed scores)	12.1 ± 1.5	.33 (.077)	−.04 (.851)
Incidental visual memory (percentiles)	60.2 ± 29.9	−.12 (.514)	.13 (.487)
Vocabulary (normed scores)	14.2 ± 2.3	.04 (.831)	.22 (.242)
Processing speed (normed scores)	11.5 ± 2.4	.13 (.482)	−.11 (.561)

Note

Degrees of freedom (*df*), 28.

Significant *p* values are indicated in bold font.

### 3.2 Imaging results

#### 3.2.1 Brain activation

**Figure 3:**
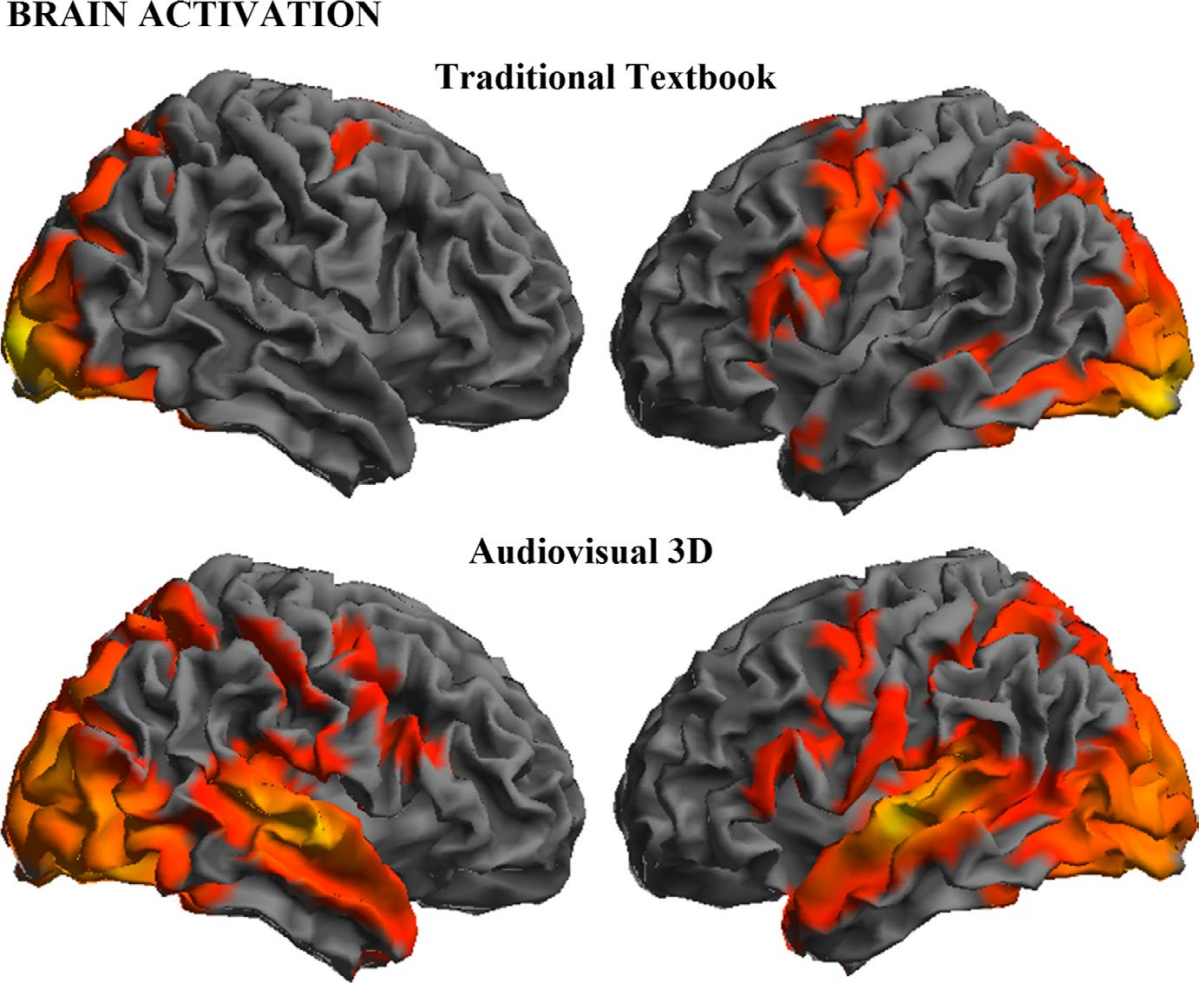
Group activation results (one‐sample *t* tests) for both traditional textbook and audiovisual‐3D lessons

Brain activation was robust in both experiments (Figure 3 and Table S1), which otherwise showed highly significant differences. During the traditional textbook lesson, the strongest activations were identified in the ventral and dorsal aspects of the visual cortex, hippocampi, and dorsal parietal association cortex including the intraparietal sulci, frontal eye fields, and left premotor cortex extending to Broca's area and Wernicke's area.

During the audiovisual‐3D lesson, all these brain regions were also activated (Figure 3, Figure S1 and Table S2), but the changes were more extensive and additionally involved other areas. Direct comparison between both data sets indeed showed significantly higher activation during the audiovisual‐3D lesson in the auditory cortex and related areas of the temporal lobe, ventral aspect of the occipito‐temporal cortex, supramarginal gyrus/parietal opercular region, and right frontal cortex (Figure 3, Figure S2 and Table S2).

In the opposite contrast, activation during the traditional textbook lesson showed significantly higher activation in the primary visual cortex at the occipital pole, right prefrontal cortex in the middle frontal gyrus, supplementary motor area, and cerebellar vermis (Figure S2 and Table S2).

#### 3.2.2 Deactivation pattern

**Figure 4:**
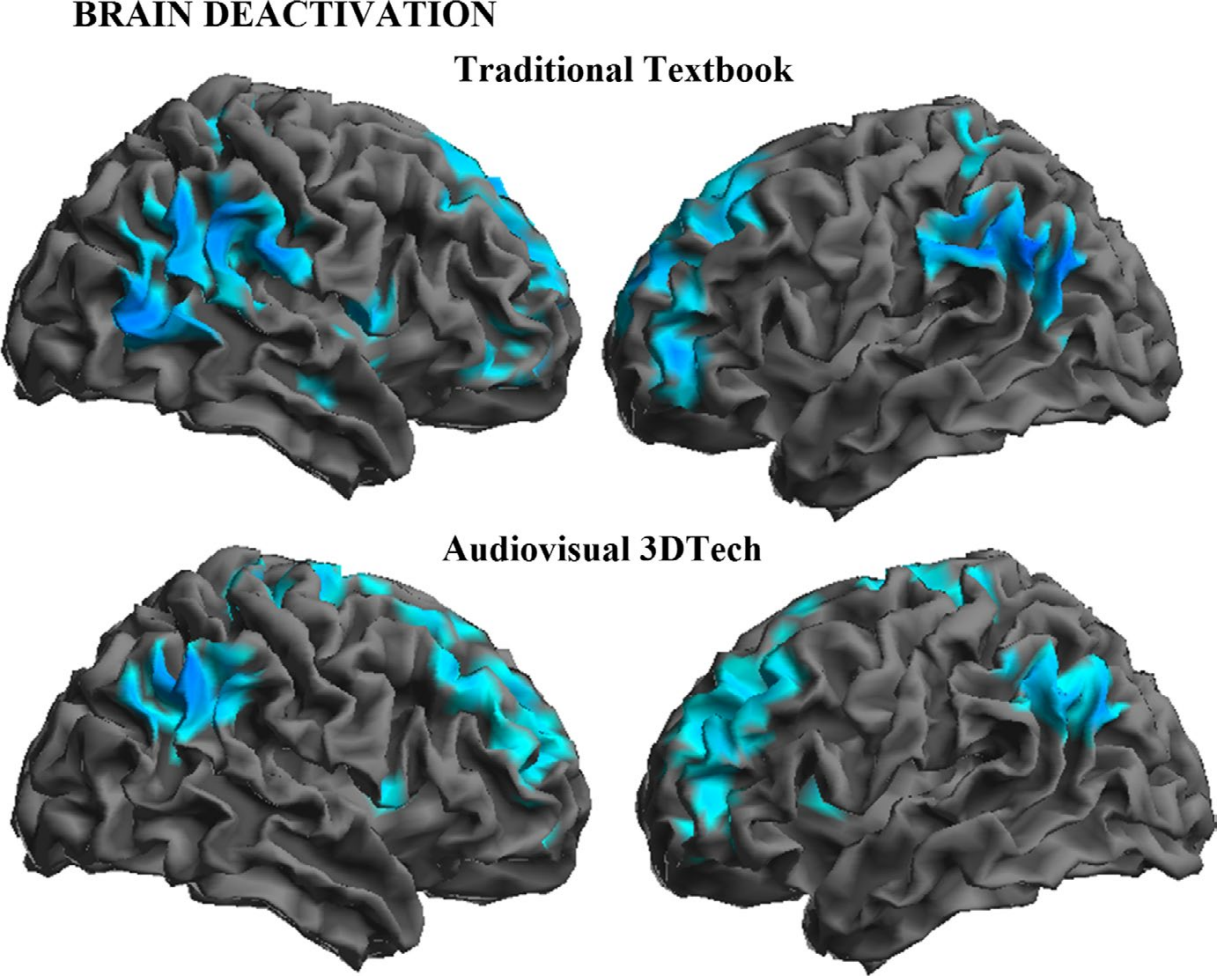
Deactivated brain areas during textbook and audiovisual‐3D lessons

The analysis of brain deactivations (i.e., higher activity during baseline than test condition) also showed robust group results (Figure 4 and Table S3). In both experiments, the pattern was dominated by changes in the dorsolateral prefrontal cortex, inferior parietal lobule (involving both the angular and supramarginal gyri), and brain medial wall structures (medial frontal and posterior cingulate cortex).

#### 3.2.3 Correlation analysis—Audiovisual‐3D lesson

**Figure 5:**
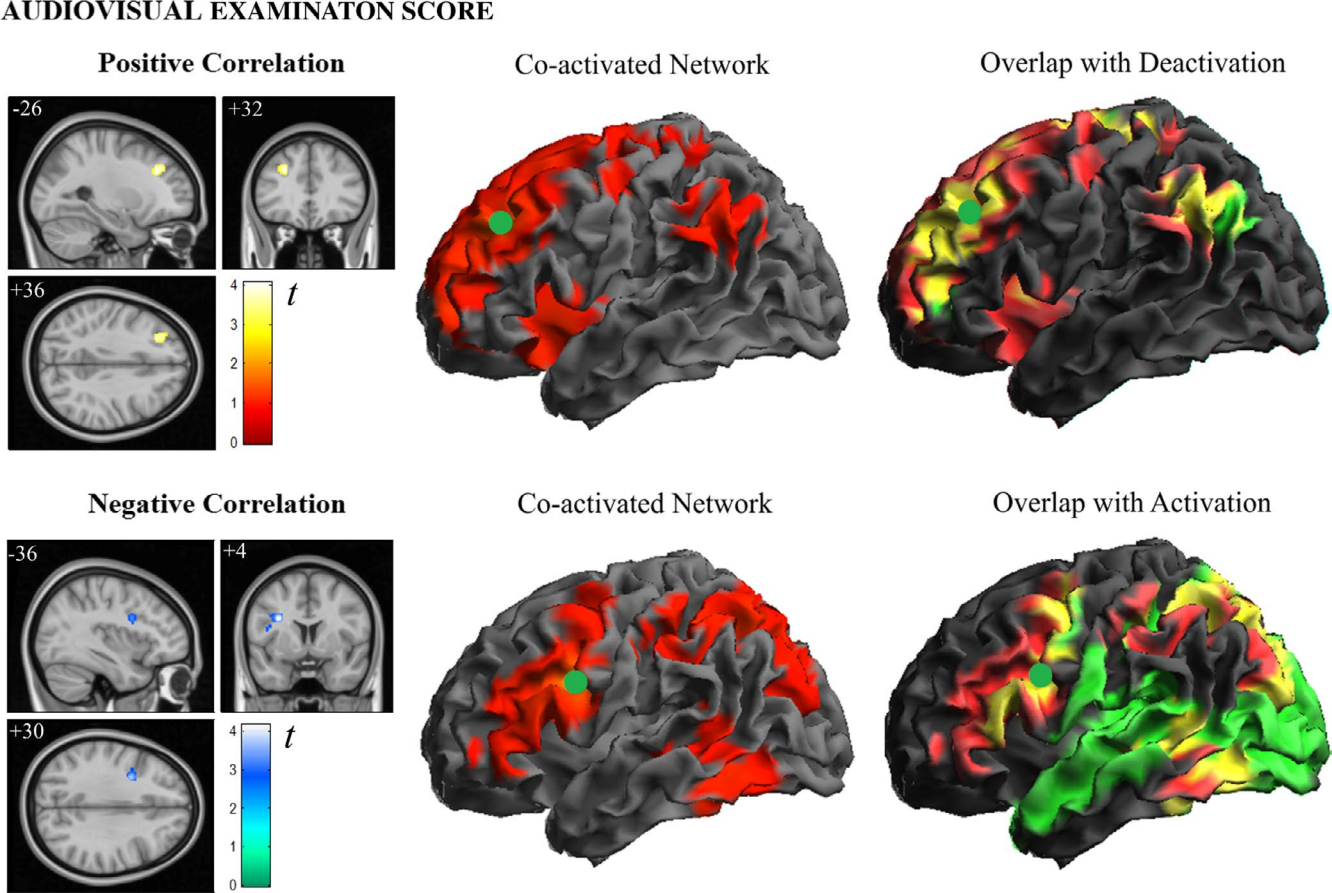
Correlation between audiovisual‐3D examination scores and brain activity during audiovisual‐3D lessons (left panels). The central panels illustrate the network of regions (seed analyses) co‐activated with the identified frontal regions. The right panels show how the regions co‐activated with the left dorsal prefrontal region overlap (yellow) with deactivated brain areas, and the regions co‐activated with the left ventral frontal region overlap (yellow) with activated brain areas. Numbers indicate MNI coordinates

**Figure 6:**
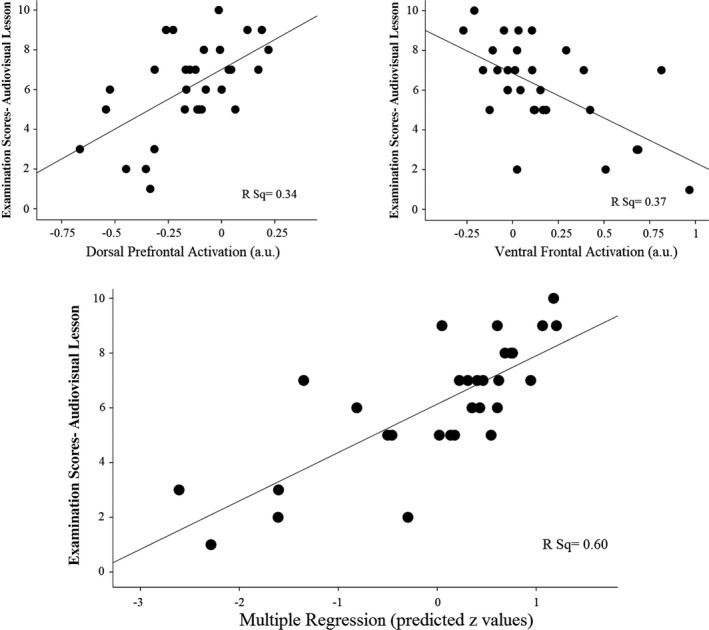
Plots showing the associations between examination scores from lessons presented with the audiovisual‐3D format and brain activity in frontal areas

Examination scores for the lessons presented with the audiovisual‐3D format showed a dual pattern of correlations with fMRI signal changes evoked during exposure to stimuli (Figure 5). Specifically, brain activity in a left dorsal prefrontal region predicted higher examination scores, whilst brain activity in a left ventral frontal region adjacent to Broca's area predicted lower scores (see also Table S4). That is, examination scores increases as dorsal prefrontal activation increases and ventral frontal activation decreases. These two opposite effects were independent and complementary in accounting for examination scores (Figure 6). That is, in a multiple regression analysis both imaging variables jointly explained 57% of audiovisual examination score variance (multiple *R* = 0.77, *r* ^2^ = 0.60, adjusted *r* ^2^ = 0.57, *F*_2,27_ = 20.1, *p* = .000005).

To illustrate the meaning of the identified associations, we mapped the networks of regions co‐activated with the frontal regions showing positive and negative correlations with examination scores (seed analyses). Overlapping displays (Figure 5) interestingly show that the regions co‐activated with the left dorsal prefrontal region during the audiovisual‐3D lesson correspond mostly to deactivated brain areas. By contrast, regions co‐activated with the left ventral frontal region adjacent to Broca's area network mostly overlap with activated regions. Therefore, higher examination scores are seen to be achieved by participants capable of recruiting, as opposed to deactivating, the prefrontal cortex during the lesson. This is evident in the plots (Figure 6), where all participants activating this region showed examination scores ≥5. Also, higher scores corresponded to individuals with less Broca's area activation, as examination scores negatively correlated with activity in this region.

To this end, we observed that the audiovisual presentation of educational material generated an extensive and robust pattern of activation that did not, however, positively predict examination scores. Examination success was instead associated with fMRI signal changes in circumscribed frontal areas.

**Figure 7:**
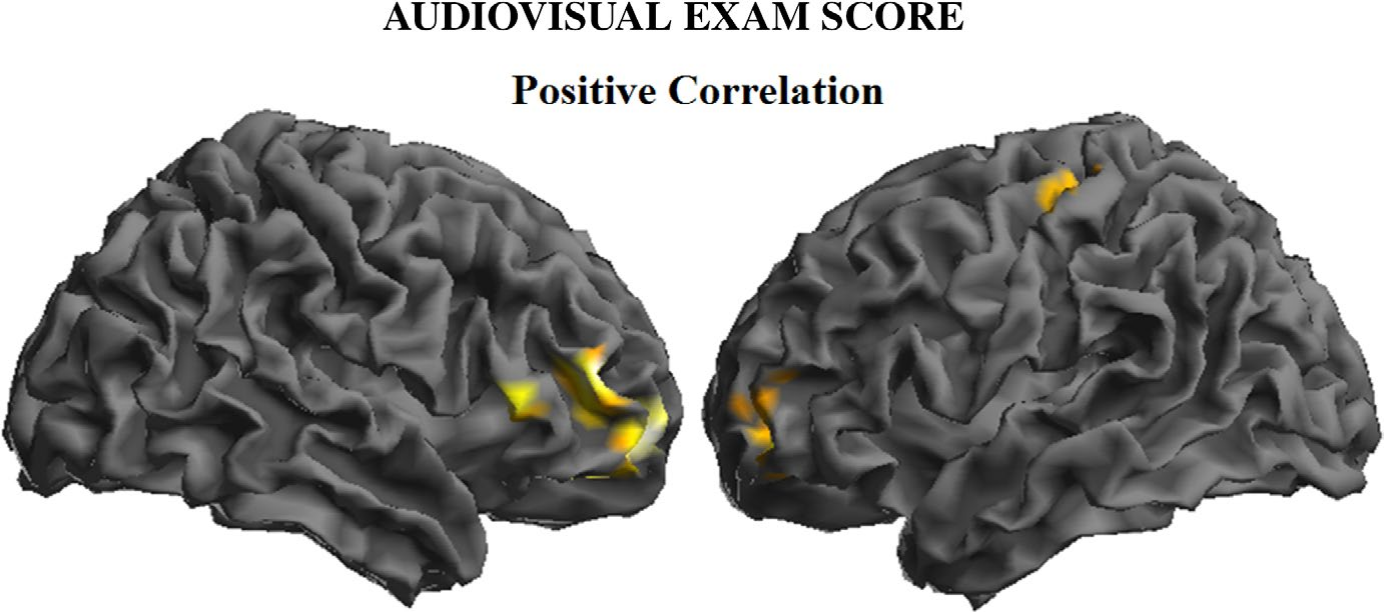
Correlation between audiovisual‐3D examination scores and brain activity immediately after exposure to audiovisual‐3D material

To characterize the temporal evolution of the learning process, we further analyzed the correlation with examination scores at four different times (beginning, middle, end of each lesson fragment, and immediately after the lesson fragment). Significant correlations were observed only upon completion of and after the lesson. During the final period, brain activity in the left dorsal prefrontal cortex once again predicted higher examination scores (Table S4). However, the most extensive correlation findings were observed after the lesson, once the audiovisual stimuli were no longer present, and involved ventral prefrontal cortex bilaterally and the left sensorimotor cortex (Figure 7 and Table S4).

#### 3.2.4 Correlation analysis—Traditional textbook

**Figure 8:**
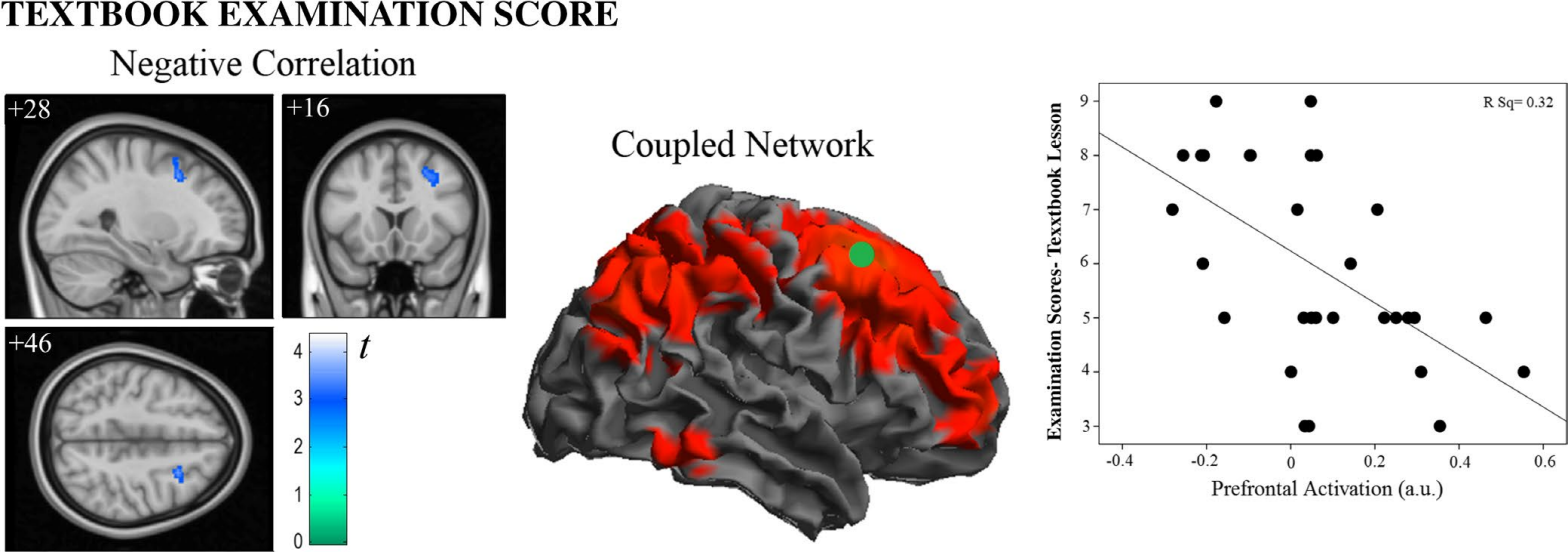
Correlation between traditional textbook scores and brain activity during traditional textbook lessons (left panel). The central panel illustrates the network of regions (seed analysis) co‐activated with the identified frontal region. The right panel shows the association between examination scores and brain activity in the frontal area. Numbers indicate MNI coordinates

Examination scores in lessons presented with the traditional textbook format showed a negative correlation with fMRI signal change in a right dorsal prefrontal region (Figure 8). This correlation interestingly shows the opposite direction to the association observed in the audiovisual‐3D analysis, which would further support the adoption of different mechanisms for learning material presented in different formats. The temporal analysis showed a significant association between examination scores and signal changes in this right dorsal prefrontal region only in the middle period (Table S4).

## 4 DISCUSSION

Educational medical topics were adapted to be presented in both traditional textbook and audiovisual‐3D lesson formats. On the basis of the test scores, students gained a similar level of knowledge in both formats. Imaging results, however, interestingly suggest that similar learning may be obtained via different brain resources. Evoked brain activity was robust during both the traditional textbook and audiovisual‐3D lessons, but a larger number of brain systems were implicated in the processing of audiovisual‐3D information, consistent with its multisensory nature and higher stimulus complexity. Significantly, learning was not associated with group mean brain activations, but was instead predicted by distinct fMRI signal changes in the frontal lobes. In the audiovisual‐3D version, examination scores positively correlated with late prefrontal cortex activity and working memory, and correlated negatively with language‐related frontal areas and verbal memory. In the traditional textbook version, the fewer results recorded suggested the opposite pattern.

During traditional textbook learning, the identified pattern of brain activation accurately reflected the expected changes for natural reading and verbal information encoding (Choi, Desai, & Henderson, 2014; Schuster, Hawelka, Hutzler, Kronbichler, & Richlan, 2016; Wandell & Le, 2017), with activation of the visual cortex, hippocampi, frontal eye fields, intraparietal sulci, Broca's area, and Wernicke's area. In addition, strong activation was identified in both the ventral and dorsal visual streams, which is consistent with neural operations related to processing object features and spatial relationships in the illustrating images presented along with the text (Goodale & Milner, 1992; Ungerleider & Haxby, 1994).

In the audiovisual version, the verbal input was aural and the visual stimulation involved more complex dynamic 3D representations. Accordingly, brain activation was more extensive and implicated the pertinent areas in each brain lobe. Additional activations were thus identified in the auditory cortex processing speech and related areas of the temporal lobe (Venezia et al., 2017), particularly involving the superior temporal sulcus likely reflecting multisensory integration (Beauchamp, Yasar, Frye, & Ro, 2008), the lateral aspect of the occipito‐temporal cortex subserving complex visual processes and motion perception (Lingnau & Downing, 2015), the supramarginal gyrus/parietal opercular region critical to cross‐modal abstractions and spatial cognition (Leichnetz, 2011), and right frontal cortex. Paradoxically, the larger number of brain resources employed to process more complex audiovisual information was not necessarily associated with greater learning, as, indeed, our participants actually obtained similar examination scores with both formats.

The issue of stimulus complexity in encoding information has previously been discussed in the context of advertising. Some data indicate that a higher sensory intensity of audio and visual features brings increased attention and cognitive processing, leading to a greater advertising impact (Langleben et al., 2009). Dynamic motion and audio features may indeed be effective in making ads more memorable (Han et al., 2015). However, stimuli wealth and complexity may otherwise exceed the capacity to assimilate information and result in reduced message retention (Langleben et al., 2009; Seelig et al., 2014; Wang et al., 2013). In our audiovisual experiment, some balance may exist between the advantages and disadvantages of providing more complex information.

The amount and complexity of the information is obviously an important factor to consider when designing audiovisual educational material, as is the tempo with which the information is presented. In our study, examination scores were predicted by brain changes at the end‐of‐lesson fragments and after‐lesson fragments when presented in the audiovisual‐3D format, which would indicate that information perception and assimilation do not temporally coincide, thus reinforcing the notion that the tempo should be adequately adjusted when planning education. By contrast, in the case of using the traditional textbook format, examination scores were predicted by brain changes at the middle‐of‐lesson fragments suggesting a different learning strategy, in which information may be more directly recorded at the perception stage.

Activity in the prefrontal cortex significantly accounted for the notable interindividual differences of our participants when assimilating didactic information presented in the audiovisual‐3D format. Successful participants activated prefrontal cortex areas typically concerned with executive functions (Fuster, 2001; Wood & Grafman, 2003). These participants also showed better working memory, which is a prototypical executive function (Diamond, 2013; Wager & Smith, 2003). On the other hand, examination scores negatively correlated with activity in language‐related cortex and verbal memory. Such a scenario would therefore indicate that audiovisual learning may benefit from executive prefrontal operations, whereas verbal processing, to some extent, interfered with information assimilation in our experiment. In other words, audiovisual learning of the presented material may be more closely related to the proper use of perceived information to integrate knowledge than to the verbal memorization of didactic material, that is to say learning through understanding as opposed to memorizing.

On the whole, our results suggest notable differences between traditional textbook and audiovisual learning of an identical amount of didactic information. The limited results obtained from the traditional textbook task analysis were in the opposite direction, with a negative correlation between examination scores and prefrontal cortex activity suggesting a lower involvement of executive operations. Moreover, the dorsal prefrontal cortex is one of the few structures showing significantly higher activation during the traditional textbook lesson (Figure S2), as a result of being less deactivated (Figure 4). The overall pattern of results (i.e., activation/deactivation and the direction of the correlation with examination scores) may therefore support the notion that prefrontal cortex activity to some extent interferes with learning during textbook lessons. Unfortunately, we found no positive correlation to complete the pattern and, thus, the analysis is less informative as to how learning takes place in such a situation. Further research is needed to support the proposal that traditional textbook learning is based more on language processing and verbal memory.

A limitation in our study concerns the generalization of results. Firstly, there is a notable difference between the duration of conventional college lessons and lesson duration in our experiment. Therefore, our results cannot be generalized at this level. Also, both textbook and audiovisual formats may potentially be more or less advantageous depending on the topics to be learned. We recorded the students' preference for the audiovisual version of the lesson, but with no clear advantage in the context of examination results. Previous studies generally coincide on reporting higher student satisfaction when using audiovisual material (Ahmad, Sleiman, Thomas, Kashani, & Ditmyer, 2016; Drapkin, Lindgren, Lopez, & Stabio, 2015; Murgitroyd, Madurska, Gonzalez, & Watson, 2015; Ozer, Govsa, & Bati, 2017; Prakash et al., 2017; Trelease, 2016), and some studies also report objective advantages based on examination scores (e.g., Ahmad et al., 2016; Drapkin et al., 2015; and revised in Trelease, 2016). As might be expected, the topic that more clearly benefits from audiovisual and 3D material is Anatomy (Ahmad et al., 2016; Drapkin et al., 2015; Trelease, 2016). Our study is also limited due to its relatively reduced cognitive evaluation and a lack of long‐term lesson learning assessments.

In conclusion, this is a novel functional MRI study characterizing the pattern of brain activity evoked during the assimilation of educational information presented in two distinct formats. Brain activation was robust in both cases, but notable differences were identified that may well suggest the advantages of either model. It is relevant, however, that the actual learning success, measured by a multiple‐choice examination, was not directly related to the most obvious brain activations. Examination scores were instead associated with fMRI signal changes in the frontal lobes. Learning during the audiovisual version was specifically predicted by a combination of more activity in the dorsal prefrontal cortex and less participation of the language‐related ventral frontal cortex. Consistently, examination scores positively correlated with performance in executive functioning and negatively with verbal memory. Overall, our results suggest that audiovisual learning relies more on the proper use of perceived information and less on basic verbal memory processes. Further research would be necessary for a more complete characterization of imaging correlates in the context of traditional textbook learning.

## CONFLICT OF INTEREST

The authors have no conflict of interest to declare.

## Data Availability

The data that support the findings of this study are available from the corresponding author upon reasonable request.
