# Supplementary material for: Brain activity during traditional textbook and audiovisual‐3D learning
Source: Brain Behav. 2019 Sep 30;9(10):e01427. doi: 10.1002/brb3.1427 (PMC6790317; doi:10.1002/brb3.1427)
Supplement: Supplementary file 1 [file BRB3-9-e01427-s001.docx]

Brain activity during traditional textbook and audiovisual-3D learning

**Supplementary Material**

**Supplementary Figures**

**
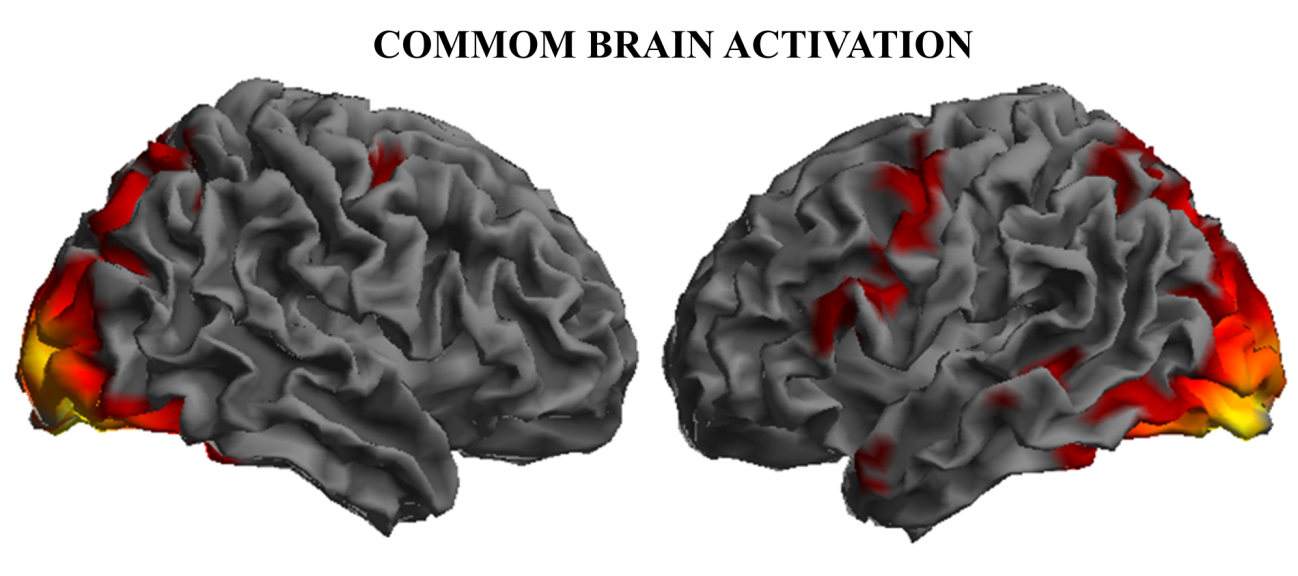
**

**Supplementary Figure 1**. Common activations across educational formats (conjunction analysis).

**
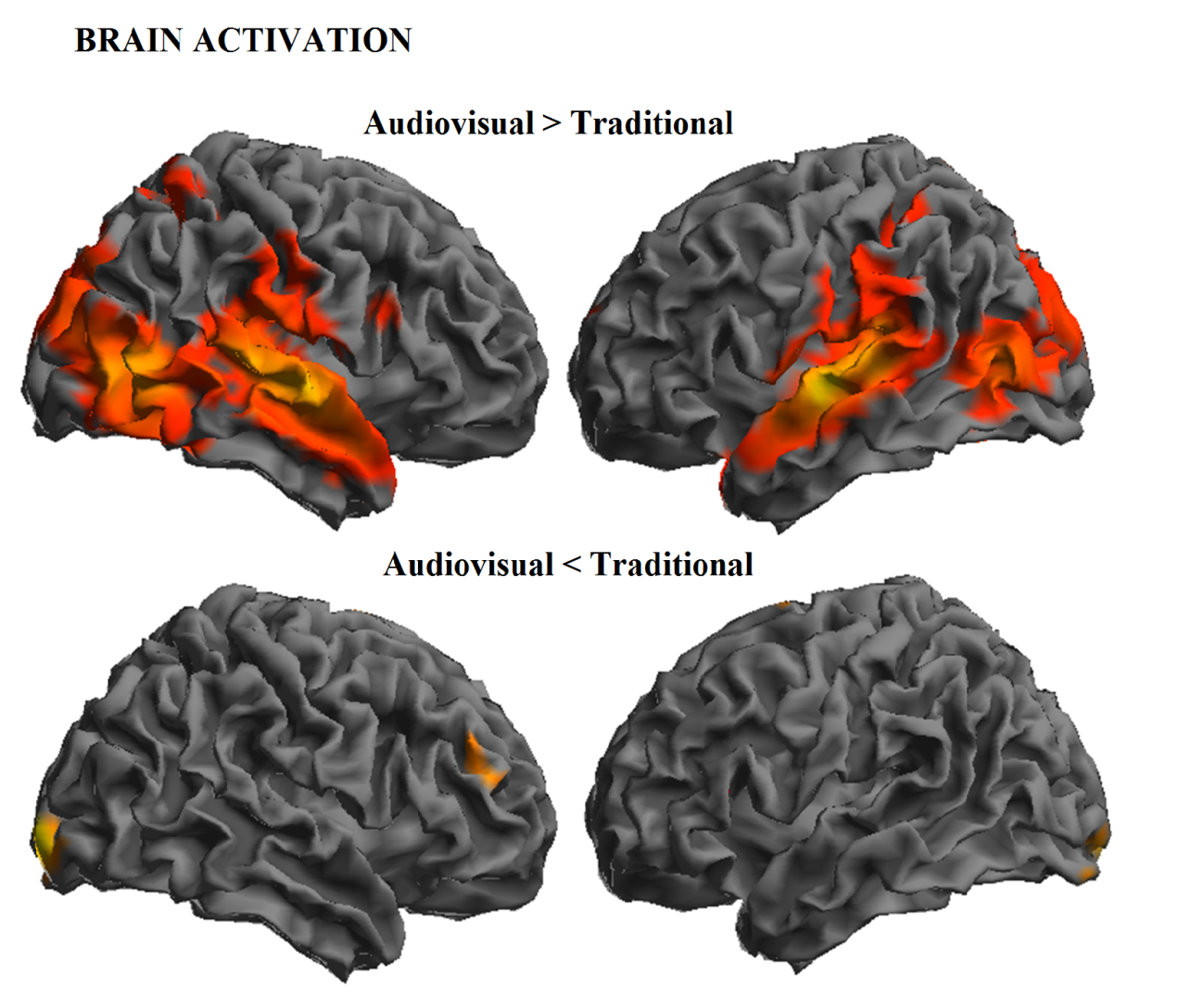
**

**Supplementary Figure 2**. Differences between traditional textbook and audiovisual lessons in evoked brain activivity.

**Supplementary Tables**

| **Supplementary Table 1.** Functional MRI results. Group activation | | | |
| --- | --- | --- | --- |
| **Traditional Textbook Lesson** | **MNI coordinates** | **t** | **p** |
| L Visual Cortex | -10 -86 -12 | 19.6 | <1e-15 |
| R Visual Cortex | 22 -102 -2 | 20.5 | <1e-15 |
| L fusiform | -34 -62 -16 | 10.7 | 2e-15 |
| R fusiform | 30 -54 -12 | 5.4 | 5e-7 |
| L Hippocampus | -24 -28 -8 | 13.0 | <1e-15 |
| R Hippocampus | 24 -26 -8 | 12.6 | <1e-15 |
| L Intraparietal Sulcus | -24 -76 38 | 7.7 | 9e-11 |
| R Intraparietal Sulcus | 26 -64 52 | 5.3 | 1e-6 |
| L Frontal Eye Field | -38 -2 62 | 5.1 | 2e-6 |
| R Frontal Eye Field | 28 -2 54 | 4.5 | 0.00001 |
| Supplementary Motor Area (anterior) | -6 4 58 | 5.4 | 5e-7 |
| Frontal Language Areas | -50 0 52 | 8.1 | 2e-11 |
| L Wernicke Area | -50 -36 -2 | 4.7 | 7e-6 |
| Brainstem | -8 -30 -8 | 7.0 | 2e-9 |
| Total brain activation 48,265 voxels, 386.1 ml | | | |
| **Audiovisual-3D Lesson** |  |  |  |
| L Visual Cortex | -10 -86 -12 | 21.5 | <1e-15 |
| R Visual Cortex | 24 -70 -10 | 22.4 | <1e-15 |
| L fusiform | -32 -60 -16 | 18.3 | <1e-15 |
| R fusiform | 28 -54 -14 | 23.0 | <1e-15 |
| L Hippocampus | -22 -28 -8 | 13.8 | <1e-15 |
| R Hippocampus | 20 -28 -4 | 14.2 | <1e-15 |
| L Intraparietal sulcus | -24 -78 38 | 10.3 | 5e-15 |
| R Intraparietal sulcus | 28 -78 30 | 14.9 | <1e-15 |
| L Frontal Eye Field | -34 -4 56 | 4.4 | 0.00002 |
| R Frontal Eye Field | 26 -2 52 | 5.1 | 2e-6 |
| Frontal Language Areas | 48 12 26 | 6.2 | 3e-8 |
| L Wernicke Area | -50 -36 -2 | 9.8 | 1e-14 |
| Brainstem | 6 -32 -8 | 10.4 | 4e-15 |
| L Lateral Frontal Cortex | -44 4 24 | 5.4 | 6e-7 |
| L Auditory Cortex- Temporal Lobe | -56 -18 -2 | 27.4 | <1e-15 |
| R Auditory Cortex- Temporal Lobe | 64 -10 -2 | 21.3 | <1e-15 |
| L Occipito-Temporal Cortex | -44 -78 6 | 13.3 | <1e-15 |
| R Occipito-Temporal Cortex | 48 -74 4 | 15.8 | <1e-15 |
| L Supramarginal gyrus/Parietal Operculum | 54 -26 14 | 8.8 | 1e-12 |
| R Supramarginal Gyrus/Parietal Operculum | 62 -26 16 | 7.5 | 2e-10 |
| Total brain activation 69,773 voxels, 558.2 ml | | | |
| MNI, Montreal Neurological Institute. Degrees of freedom (df), 29. | | | |

| **Supplementary Table 2.** Functional MRI results. Differences between educational formats | | | | | | | | |
| --- | --- | --- | --- | --- | --- | --- | --- | --- |
| **Traditional Textbook < Audiovisual-3D** | **MNI coordinates** | | **Effect size*** | | **t** | | **p** | |
| L Auditory Cortex- Temporal Lobe | -56 -18 -2 | | 7.4 | | 19.9 | | <1e-15 | |
| R Auditory Cortex- Temporal Lobe | 64 -10 -2 | | 6.0 | | 16.1 | | <1e-15 | |
| L Occipito-Temporal Cortex | -48 -76 6 | | 3.7 | | 10.0 | | 1e-14 | |
| R Occipito-Temporal Cortex | 50 -72 4 | | 5.3 | | 14.4 | | <1e-15 | |
| L Supramarginal Gyrus/Parietal Operculum | -60 -30 34 | | 1.7 | | 4.5 | | 0.00002 | |
| R Supramarginal Gyrus/Parietal Operculum | 60 -26 22 | | 2.3 | | 6.1 | | 7e-8 | |
| R Lateral Frontal Cortex | 54 14 22 | | 1.4 | | 3.7 | | 0.0002 | |
| Total brain differences 30,700 voxels, 245.6 ml | | | | | | | | |
| **Traditional Textbook > Audiovisual-3D** |  | |  | |  | |  | |
| L Primary Visual Cortex | -28 -100 -10 | | 1.9 | | 5.2 | | 2e-6 | |
| R Primary Visual Cortex | 24 -104 -4 | | 2.5 | | 6.7 | | 5e-9 | |
| R Prefrontal Cortex | 46 46 18 | | 1.3 | | 3.4 | | 0.0006 | |
| Supplementary Motor Area | -6 2 58 | | 1.2 | | 3.2 | | 0.001 | |
| Cerebellum | 2 -78 -30 | | 1.7 | | 4.6 | | 0.00001 | |
| Total brain differences 2,120 voxels, 17.0 ml | | | | | | | | |
| Common activations across educational formats (conjunction analysis) | | | | | | | | |
| L Visual Cortex | | -10 -86 -12 | | 7.3 | | 19.6 | | <1e-15 |
| R Visual Cortex | | 22 -98 2 | | 5.0 | | 13.4 | | <1e-15 |
| L fusiform | | -34 -62 -16 | | 4.0 | | 10.7 | | 2e-15 |
| R fusiform | | 34 -62 -16 | | 2.5 | | 6.6 | | 6e-9 |
| L Hippocampus | | -24 -28 -8 | | 4.8 | | 13.0 | | <1e-15 |
| R Hippocampus | | 24 -28 -6 | | 4.6 | | 12.4 | | <1e-15 |
| L Intraparietal Sulcus | | -24 -74 32 | | 2.8 | | 7.6 | | 1e-10 |
| R Intraparietal Sulcus | | 26 -64 52 | | 2.0 | | 5.3 | | 1e-6 |
| L Frontal Eye Field | | -38 -2 62 | | 1.7 | | 4.5 | | 0.00001 |
| R Frontal Eye Field | | 28 -2 54 | | 1.7 | | 4.5 | | 0.00001 |
| Frontal Language Areas | | -44 4 24 | | 2.0 | | 5.4 | | 7e-7 |
| L Wernicke Area | | -50 -36 -2 | | 1.7 | | 4.7 | | 7e-6 |
| Brainstem | | -8 -30 -8 | | 2.6 | | 7.0 | | 2e-9 |
| Total brain common activation, 38,400 voxels,307.2 ml | | | | | | | | |
| MNI, Montreal Neurological Institute. Degrees of freedom (df), 29. * Cohen’s d. | | | | | | | | |

| **Supplementary Table 3.** Functional MRI results. Group deactivation | | | |
| --- | --- | --- | --- |
| **Traditional Textbook Lesson** | **MNI coordinates** | **t** | **p** |
| L Prefrontal Cortex | -24 26 40 | 5.1 | 2e-6 |
| R Prefrontal Cortex | 14 58 8 | 5.8 | 5e-8 |
| Anterior Cingulate Cortex/Medial Frontal | -10 42 -6 | 6.8 | 4e-9 |
| Posterior Cingulate Cortex/Precuneus | -10 -56 34 | 4.9 | 3e-6 |
| L Angular/Supramarginal Gyrus | -58 -64 32 | 8.2 | 2e-11 |
| R Angular/Supramarginal Gyrus | 60 -64 8 | 7.3 | 6e-10 |
| R Insula/operculum | 54 0 8 | 4.9 | 4e-6 |
| Total brain deactivation 20,668 voxels, 165.3 ml | | | |
| **Audiovisual-3D Lesson** |  |  |  |
| L Prefrontal Cortex | -32 26 36 | 5.5 | 5e-7 |
| R Prefrontal Cortex | 20 42 32 | 5.4 | 7e-7 |
| L Motor Cortex (sensorimotor) | -20 -32 62 | 5.0 | 3e-6 |
| R Motor Cortex (sensorimotor) | 22 -32 62 | 6.2 | 3e-8 |
| Posterior Cingulate Cortex/Precuneus | -6 -64 36 | 5.2 | 2e-6 |
| L Angular Gyrus | -50 -76 34 | 7.7 | 1e-10 |
| R Angular Gyrus | 52 -54 44 | 7.5 | 2e-10 |
| L Insula | -30 10 8 | 4.0 | 0.00008 |
| R Insula/operculum | 54 6 6 | 4.2 | 0.00005 |
| Total brain deactivation 18,777 voxels, 150.2 ml | | | |
| MNI, Montreal Neurological Institute. Degrees of freedom (df), 29. | | | |

| **Supplementary Table 4**. Functional MRI results. Correlation analysis | | | | | |  |
| --- | --- | --- | --- | --- | --- | --- |
|  | **Cluster size**  **Voxels (ml)** | **MNI**  **coordinates** | **r** | **t** | **p** | |
| **AUDIOVISUAL-3D LESSON** |  |  |  |  |  | |
| **Stimulus exposure- Total period** |  |  |  |  |  | |
| Positive Correlation with Exam scores |  |  |  |  |  | |
| L Dorsal Prefrontal Cortex | 133 (1.1) | -28 30 32 | 0.6 | 4.1 | 0.0002 | |
| Negative Correlation with Exam scores |  |  |  |  |  | |
| L Ventral Frontal Cortex | 126 (1.0) | -32 4 26 | 0.6 | 4.2 | 0.0001 | |
| **Last stimulus exposure period** |  |  |  |  |  | |
| Positive Correlation with Exam scores |  |  |  |  |  | |
| L Dorsal Prefrontal Cortex | 165 (1.3) | -28 30 32 | 0.6 | 4.3 | 0.0001 | |
| L Ventral Prefrontal Cortex | 159 (1.3) | -26 48 2 | 0.6 | 3.9 | 0.0005 | |
| **Post stimulus exposure** |  |  |  |  |  | |
| Positive Correlation with Exam scores |  |  |  |  |  | |
| R Ventral Prefrontal Cortex | 1030 (8.2) | 30 60 -8 | 0.7 | 5.0 | 0.00001 | |
| L Ventral Prefrontal Cortex | 316 (2.5) | -30 60 2 | 0.6 | 4.1 | 0.0002 | |
| L Sensorimotor Cortex | 168 (1.3) | -44 -22 56 | 0.6 | 3.7 | 0.0005 | |
| **TRADITIONAL TEXTBOOK LESSON** |  |  |  |  |  | |
| **Stimulus exposure- Total period** |  |  |  |  |  | |
| Negative Correlation with Exam scores |  |  |  |  |  | |
| R Dorsal Prefrontal Cortex | 135 (1.1) | 26 14 48 | 0.6 | 3.6 | 0.0006 | |
| **Middle stimulus exposure period** |  |  |  |  |  | |
| Negative Correlation with Exam scores |  |  |  |  |  | |
| R Dorsal Prefrontal Cortex | 120 (1.0) | 32 16 44 | 0.6 | 4.1 | 0.0002 | |
| MNI, Montreal Neurological Institute. Degrees of freedom (df), 28. | | | | | |  |
